# Platform of food allergens in Argentina

**DOI:** 10.1186/2045-7022-1-S1-P16

**Published:** 2011-08-12

**Authors:** Claudia Gonzalez, María Cristina Lopez

**Affiliations:** 1 National Institute of Agricultural Technology, Strategy Area of Food Technology, Buenos Aires, Argentina; 2 National Institute of Industrial Technology, Cereals and Oilseeds, San Martín, Argentina

## 

The Platform of Food Allergens was formally created in Argentina in April 2009. It is a multidisciplinary discussion forum formed by professionals from different government institutions such as the National Institute of Agricultural Technology (INTA), National Institute of Industrial Technology (INTI), National Institute of Foods (INAL); School of Pharmacy and Biochemistry (Buenos Aires University), School of Exact Sciences (La Plata University); members of the Argentine Allergy and Clinical Immunology Association and the "Forum of parents of allergic children", medical doctors from the Children's Hospital "Ricardo Gutierrez", and an important number of food industry companies. The aims of the Platform, as a non profit organization, are:

• To anticipate future demands, since this issue is still a vacancy in Argentina.

• To collaborate with the regulatory authorities in enhancing the legal frame of the declaration of food allergens in prepackaged foods.

• To provide the food allergic population with information and guarantees at the time of the selection of foods.

• To provide the food industry with information and guidance for food allergen management. In order to achieve these challenges, the platform was organized in four working groups:

"Management of Food Allergens in the Food Industry" "Detection Methods of Allergens in Foods" "Legal Frame of Food Allergens" "Clinical Issues of Food Allergies" These four groups work independently and also in mutual collaboration, in order to cover the entire spectrum of requirements. The objective of this presentation is to show some of the activities and achievements, which these working groups have obtained since the Argentinean Platform was created.

